# Nucleic Acid Delivery by Solid Lipid Nanoparticles Containing Switchable Lipids: Plasmid DNA vs. Messenger RNA

**DOI:** 10.3390/molecules25245995

**Published:** 2020-12-18

**Authors:** Itziar Gómez-Aguado, Julen Rodríguez-Castejón, Mónica Vicente-Pascual, Alicia Rodríguez-Gascón, Ana del Pozo-Rodríguez, María Ángeles Solinís Aspiazu

**Affiliations:** 1 Pharmacokinetic, Nanotechnology and Gene Therapy Group (PharmaNanoGene), Faculty of Pharmacy, Centro de investigación Lascaray ikergunea, University of the Basque Country UPV/EHU, Paseo de la Universidad 7, 01006 Vitoria-Gasteiz, Spain; itziar.gomez@ehu.eus (I.G.-A.); julen.rodriguez@ehu.eus (J.R.-C.); monica.vicente@ehu.eus (M.V.-P.); alicia.rodriguez@ehu.eus (A.R.-G.); 2 Bioaraba, Pharmacokinetic, Nanotechnology and Gene Therapy Group (PharmaNanoGene), 01006 Vitoria-Gasteiz, Spain

**Keywords:** gene therapy, solid lipid nanoparticles, pDNA, mRNA, cationic lipid, ionizable lipid, intracellular disposition, long-term storage

## Abstract

The development of safe and effective nucleic acid delivery systems remains a challenge, with solid lipid nanoparticle (SLN)-based vectors as one of the most studied systems. In this work, different SLNs were developed, by combination of cationic and ionizable lipids, for delivery of mRNA and pDNA. The influence of formulation factors on transfection efficacy, protein expression and intracellular disposition of the nucleic acid was evaluated in human retinal pigment epithelial cells (ARPE-19) and human embryonic kidney cells (HEK-293). A long-term stability study of the vectors was also performed. The mRNA formulations induced a higher percentage of transfected cells than those containing pDNA, mainly in ARPE-19 cells; however, the pDNA formulations induced a greater protein production per cell in this cell line. Protein production was conditioned by energy-dependent or independent entry mechanisms, depending on the cell line, SLN composition and kind of nucleic acid delivered. Vectors containing 1,2-dioleoyl-3-trimethylammonium-propane (DOTAP) as unique cationic lipid showed better stability after seven months, which improved with the addition of a polysaccharide to the vectors. Transfection efficacy and long-term stability of mRNA vectors were more influenced by formulation-related factors than those containing pDNA; in particular, the SLNs containing only DOTAP were the most promising formulations for nucleic acid delivery.

## 1. Introduction

With the development of RNA-based products, the potential of therapeutic targets has expanded significantly. The United States Food and Drug Administration (FDA) has authorized a number of RNA drugs targeted to various human diseases. These RNA drugs include aptamer RNAs (e.g., pegaptanib), antisense oligonucleotides (ASOs) or antisense RNAs (asRNAs) (e.g., mipomersen, eteplirsen, nusinersen, inotersen and golodirsen), and short interfering RNAs (siRNAs) (e.g., patisiran and givosiran) [1]. Additionally, the development and use of messenger RNA (mRNA) also has broad potential for the treatment of human infections, cancers and genetic disorders, by means of protein replacement therapy, vaccination and antibody therapy [2,3]. Moreover, mRNAs are a promising tool for use in the expanding area of genome editing [4,5]. Classically, all of these therapeutic applications have been addressed through the administration of plasmid DNA (pDNA). However, mRNA possesses specific characteristics that make it a promising alternative to pDNA [6]. From a safety point of view, mRNA is not integrated into the cell genome, and the risk of carcinogenesis and mutagenesis is much lower than with pDNA. Regarding the protein expression, it is faster and temporary (actually, the expression can be detected within 1 h after transfection [7,8]). An additional advantage is that the production and manufacturing is simpler than that of DNA, and it can be standardized while maintaining reproducibility. Finally, from a delivery point of view, mRNA must only reach the cytoplasm to interact with the cellular translation machinery, whereas pDNA requires entry into the nucleus, which is one of the most limiting steps for transfection.

Many mRNA-based therapies have already entered clinical trials [9,10,11,12]; however, the construction of safe and effective delivery systems remains a challenge. The desired pharmacological effect will be achieved only if a sufficient number of RNA molecules access the intracellular environment. An optimal delivery system should avoid the degradation of the mRNA by serum RNases and allow it to pass through the target cell membranes. Significant attention is focused on the development of biocompatible carriers, such as lipid nanoparticles (LNPs), polymer nanoparticles, complexes formed by proteins or peptides and mRNA, among others. In particular, lipid-based nanomaterials are currently one of the most promising biomaterials that mediate effective mRNA delivery, with LNPs as the most advanced vector and the delivery vehicle of choice for mRNA [13]. Solid lipid nanoparticles (SLNs) are a kind of LNP that is regarded as one of the most versatile and effective non-viral vectors in gene therapy [14]. Our research group has developed SLNs based on cationic lipids for pDNA administration that have proven to be effective in different in vitro and in vivo applications, including a murine model of retinoschisis, a degenerative retinal disease [15,16]. SLNs are formed by well-tolerated physiological lipids as solid lipid core matrix dispersed in aqueous solution stabilized by surfactants, which usually includes cationic lipids that allows electrostatic interactions with nucleic acids [17,18]. One of the advantages of this versatile delivery system is that it can be reformulated depending of the features of the genetic material to be delivered. In this sense, nowadays, nanocarriers containing ionizable or switchable cationic lipids are among the leading delivery system candidates with promising applications for mRNA delivery [19]. Cationic lipids are a critical component of these lipidic systems, whose function is to interact with the mRNA leading to the formation of a lipoplex. The traditional cationic lipid 1,2-dioleoyl-3-trimethylammonium-propane (DOTAP) is one of the most used for mRNA delivery. This lipid is completely protonated at pH 7.4, so it has been postulated that, for successful transfection, high energy is required for the separation of nucleic acid from the lipoplex. Thus, to improve its efficacy in nucleic acid delivery, DOTAP should be combined with a helper lipid [20]; in fact, it is frequently combined with the zwitterionic lipid DOPE (dioleoylphosphatidylethanolamine). The use of ionizable lipids, such as 1,2-dioleoyl-3-dimethylammonium-propane (DODAP) and N-(4-carboxybenzyl)-N,N-dimethyl-2,3-bis(oleoyloxy)propan-1-aminium (DOBAQ), is a more recent strategy; these lipids are neutral at physiological pH but become protonated in the acidic environment of the endosome. The electrostatic interactions between the lipids of the endosomal membranes and the ionizable cationic lipids promote membrane lytic non-bilayer structures such as the hexagonal HII phase, facilitating the endosomal scape and the intracellular nucleic acid delivery [21,22].

The aim of the present work was to evaluate the influence of formulation factors in the efficacy of SLNs as pDNA or mRNA delivery systems. Different non-viral vectors formulated with SLNs composed by combinations of cationic lipids and ionizable cationic lipids were characterized in terms of size, polydispersity index and zeta potential, as well as their capacity to bind protect and release the nucleic acids. The transfection efficacy was assessed in vitro in two cell models (ARPE-19 and HEK-293), and the cellular uptake and intracellular disposition of the genetic material were also evaluated. Finally, a long-term stability study of the vectors was performed.

## 2. Results

### 2.1. Size, Polydispersity Index and ζ-Potential of SLNs and Vectors

Table 1 shows the mean diameter, polydispersity index (PDI) and ζ-potential of the SLNs prepared with different combinations of cationic and ionizable lipids. SLN1, SLN2 and SLN4 showed similar particle size ranging from 185 to 211 nm, PDI values lower than 0.4 and superficial charge higher than +40 mV. However, SLN3, prepared without the cationic lipid DOTAP, presented a particle size higher than 400 nm, PDI higher than 0.5 and negative surface charge of −28 mV. Considering these results, SLN3 formulation was discarded for preparing vectors.

Table 2 shows the size, PDI and ζ-potential of the SLN-based vectors containing either CleanCap™ EGFP mRNA (5moU) or pcDNA3-EGFP plasmid, which encode the enhanced green fluorescent protein (EGFP). All vectors contained protamine (P), and some of them a polysaccharide, dextran (DX) or hyaluronic acid (HA). Particle size ranged from 176 to 349 nm, PDIs was lower than 0.4 and surface charge ranged from +18 to +45 mV.

No significant difference in particle size, PDI or ζ-potential was observed when SLNs were labeled with Nile Red (data not shown).

### 2.2. Transmission Electron Microscopy (TEM) Images

**Figure 1:**
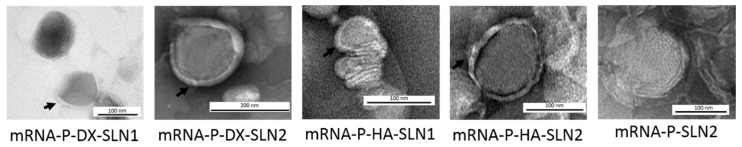
Images of mRNA combined with P, a polysaccharide (DX or HA) and SLN1 or SLN2, acquired by TEM. Black arrows indicate an external layer surrounding the electro-dense nucleus of the vectors.

Figure 1 shows TEM photographs of mRNA-vectors. In all cases, except for mRNA-P-SLN2, the structure appears compact and homogenous, with an external layer (black arrows) surrounding the electro-dense nucleus. When mRNA was complexed with P and SLN2 (without polysaccharide) (see last image, on the right, of Figure 1), a characteristic multilamellar internal structure was observed.

### 2.3. Binding, Protection and Release of Nucleic Acids

**Figure 2:**
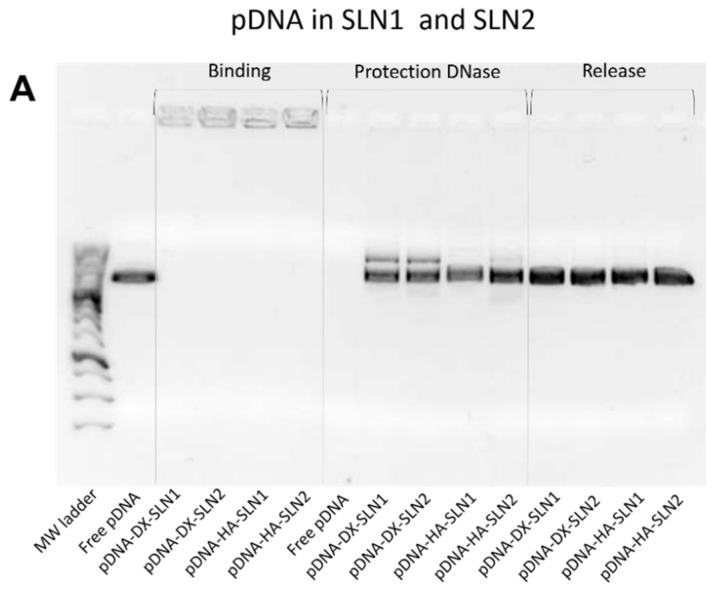

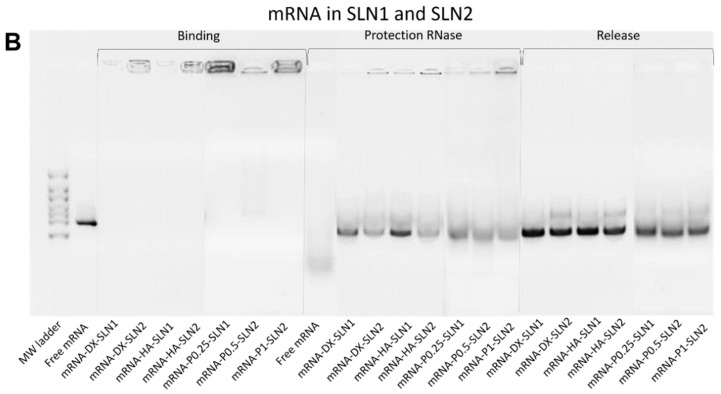
Binding, protection and release capacity of pDNA- and mRNA-based vectors: (**A**) pDNA-SLN1 and pDNA-SLN2 vectors; (**B**) mRNA-SLN1 and mRNA-SLN2 vectors.

Figure 2 shows the ability of SLN1 and SLN2 vectors to bind, protect and release the pDNA (Figure 2A) and mRNA (Figure 2B), respectively.

Regarding the binding capacity, in both gels the absence of bands on the corresponding lanes and the presence of pDNA (Figure 2A) and mRNA (Figure 2B) on the loading wells indicate that the nucleic acid was unable to migrate through the gel, and, therefore, it was completely bound to the vector.

Moreover, all pDNA-based formulations were able to protect the plasmid when treated with DNase I, while free pDNA was totally degraded. After the treatment with SDS, pDNA was able to migrate from the loading wells, which demonstrates its ability to be released from SLN1 and SLN2 vectors.

In the case of mRNA-based systems, differences in the protection were detected depending on the composition of the vector. Bands corresponding to the vectors prepared with SLN1 and DX or HA were more intense, which indicates a higher protection degree of the nucleic acid. All formulations were capable to release the mRNA after the treatment with SDS.

The evaluation of the formulations prepared with SLN4 revealed a low capacity to protect the pDNA (Supplementary Materials Figure S1), and, therefore, they were excluded for following experiments.

### 2.4. Cell Culture Studies

#### 2.4.1. Transfection Efficacy and Cell Viability in ARPE-19 Cells

**Figure 3:**
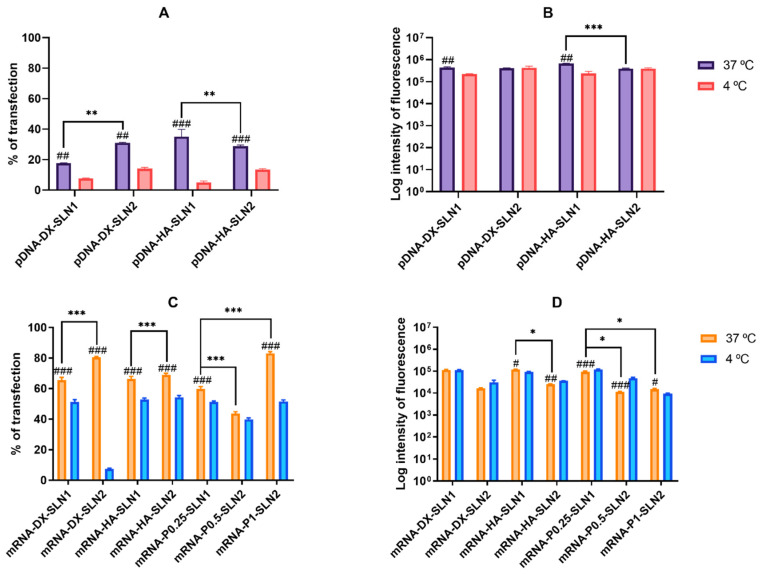
Percentage of transfected cells and intensity of fluorescence of ARPE-19 cells after incubation with SLN1- and SLN2-based vectors at 37 and 4 °C. Data are expressed as mean ± standard deviation; *n* = 3. (**A**) Percentage of transfected ARPE-19 cells 72 h after treatment with pDNA vectors. (**B**) Intensity of fluorescence of transfected ARPE-19 cells 72 h after treatment with pDNA vectors. (**C**) Percentage of transfected ARPE-19 cells 48 h after treatment with mRNA vectors. (**D**) Intensity of fluorescence of transfected ARPE-19 cells 48 h after treatment with mRNA vectors. ^#^ *p* < 0.05 with respect to the same vector at 4 °C. ^##^ *p* < 0.01 with respect to the same vector at 4 °C. ^###^ *p* < 0.001 with respect to the same vector at 4 °C. * *p* < 0.05 with respect to the other formulation. ** *p* < 0.01 with respect to the other formulation. *** *p* < 0.001 with respect to the other formulation.

Figure 3 shows the percentage of transfected cells and intensity of fluorescence of ARPE-19 cells treated with SLN1- and SLN2-based vectors at 37 and 4 °C.

At 37 °C, pDNA-based vectors (Figure 3A) induced lower percentage of transfected cells than mRNA-based vectors (Figure 3C). However, the vectors prepared with pDNA (Figure 3B) provided higher fluorescence intensities than those containing mRNA (Figure 3D).

Regarding pDNA vectors, at 37 °C, the percentage of transfected cells (Figure 3A) observed was 35% and 20% for pDNA-HA-SLN1-and pDNA-DX-SLN1, respectively. SLN2-based formulations presented a 30% of transfected cells regardless the polysaccharide used. In terms of intensity (Figure 3B), pDNA-HA-SLN1 showed the highest value (*p* < 0.001). At 4 °C, the percentage of transfected cells decreased with all formulations, ranging from 5% to 14% (Figure 3A), although the fluorescence intensity only diminished in the case of SLN1-based vectors (Figure 3B).

At 37 °C, although the highest values of transfected cells with mRNA-based vectors were observed with SLN2 formulations; in particular, for mRNA-DX-SLN2 and mRNA-P1-SLN2 (Figure 3C), the vectors prepared with SLN1 were much more effective in terms of intensity of fluorescence (Figure 3D). At 4 °C, the percentage of transfected cells decreased, especially in the case of mRNA-DX-SLN2 (Figure 3C).

Cell viability of ARPE-19 cells treated with the SLN-based vectors was around 100% at either 37 or 4 °C.

#### 2.4.2. Transfection Efficacy and Cell Viability in HEK-293 Cells

**Figure 4:**
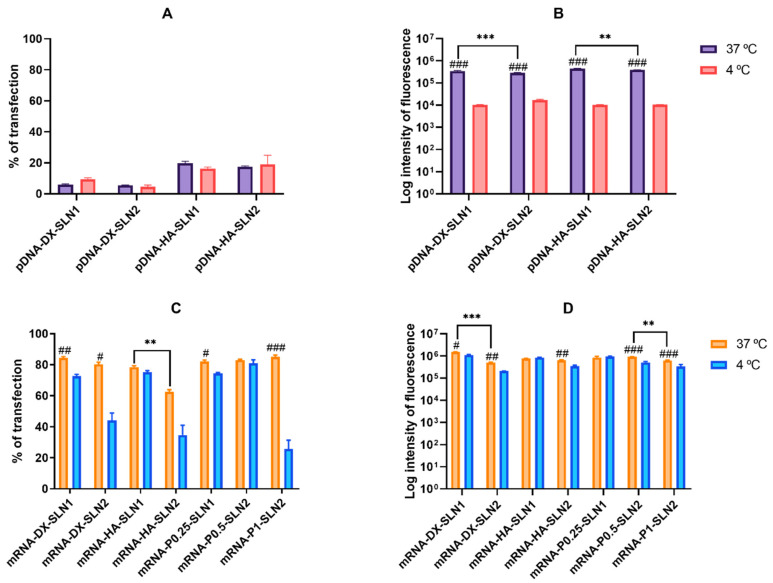
Percentage of transfected cells and intensity of fluorescence of HEK-293 cells after incubation with SLN1- and SLN2-based vectors at 37 and 4 °C. Data are expressed as mean ± standard deviation; *n* = 3. (**A**) Percentage of transfected HEK-293 cells 72 h after treatment with pDNA vectors. (**B**) Intensity of fluorescence of transfected HEK-293 cells 72 h after treatment with pDNA vectors. (**C**) Percentage of transfected HEK-293 cells 48 h after treatment with mRNA vectors. (**D**) Intensity of fluorescence of transfected HEK-293 cells 48 h after treatment with mRNA vectors. ^#^ *p* < 0.05 with respect to the same vector at 4 °C. ^##^ *p* < 0.01 with respect to the same vector at 4 °C. ^###^ *p* < 0.001 with respect to the same vector at 4 °C. ** *p* < 0.01 with respect to the other formulation. *** *p* < 0.001 with respect to the other formulation.

Figure 4 shows the percentage of transfected cells and intensity of fluorescence of HEK-293 cells treated with SLN1- and SLN2-based vectors at 37 and 4 °C.

The percentage of transfected cells with pDNA-based vectors ranged from 5% to 20%, at 37 °C (Figure 4A), and, as in ARPE-19 cells, it was significantly lower than that achieved with mRNA-based vectors (60%–80%) (Figure 4C). Nevertheless, contrary to that observed in ARPE-19 cells, the vectors prepared with mRNA also provided the highest fluorescence intensities (Figure 4D).

In the case of pDNA-based vectors, at 37 °C, the inclusion of HA in the formulation provided the highest percentage of transfection (*p* < 0.01). At 4 °C, the percentage of transfected cells was similar to that observed at 37 °C, but there was a drastic reduction in the fluorescence intensity for all formulations (Figure 4B).

The mRNA-based vectors showed a similar percentage of transfected cells at 37 °C, over 80%, except for mRNA-HA-SLN2, which presented a percentage of 60% (Figure 4C). Intensity of fluorescence was higher for SLN1 formulations than for SLN2 (Figure 4D). At 4 °C, the percentage of transfected cells decreased significantly with all formulations, except for mRNA-HA-SLN1 and mRNA-P0.5-SLN2 (Figure 4C), and the intensity of fluorescence decreased for SLN2 formulations and mRNA DX-SLN1 (Figure 4D).

Cell viability was close to 100% and over 85% for pDNA and mRNA-based vectors, respectively, at either 37 or 4 °C.

#### 2.4.3. Cellular Uptake in ARPE-19 Cells

**Figure 5:**
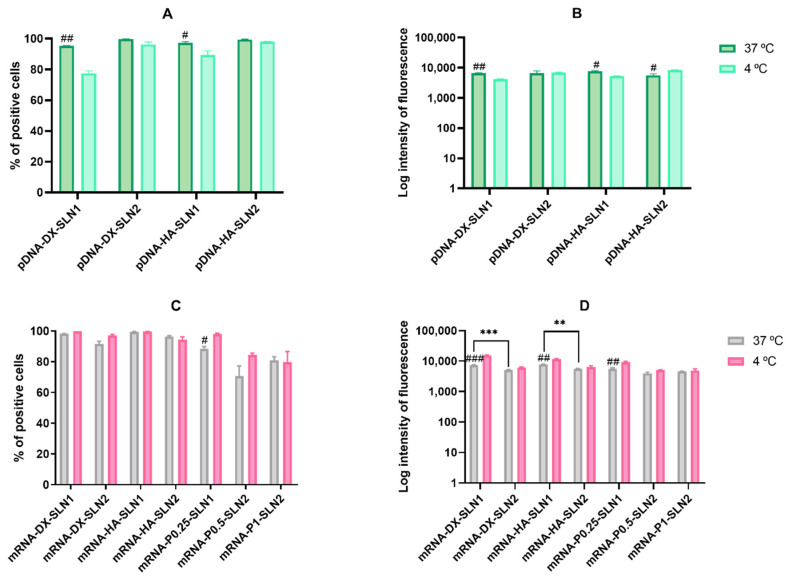
Flow cytometry analysis of cellular uptake of vectors using Nile Red–labeled pDNA- and mRNA-SLNs in ARPE-19 cells at 37 and 4 °C. Data are expressed as mean ± standard deviation; *n* = 3. (**A**) Percentage of Nile Red positive ARPE-19 cells 2 h after the treatment with pDNA vectors. (**B**) Intensity of fluorescence of Nile Red positive ARPE-19 cells 2 h after the treatment with pDNA vectors. (**C**) Percentage of Nile Red positive ARPE-19 cells 2 h after the treatment with mRNA vectors. (**D**) Intensity of fluorescence of Nile Red positive ARPE-19 cells 2 h after the treatment with mRNA vectors. ^#^ *p* < 0.05 with respect to the same vector at 4 °C. ^##^ *p* < 0.01 with respect to the same vector at 4 °C. ^###^ *p* < 0.001 with respect to the same vector at 4 °C. ** *p* < 0.01 with respect to the other formulation. *** *p* < 0.001 with respect to the other formulation.

Figure 5 shows the efficacy of cell internalization in ARPE-19 cells at 37 and 4 °C. For this study, cells were treated with the pDNA- and mRNA-based vectors labeled with Nile Red, and the uptake was measured 2 h later. The histograms showing the cellular uptake are included in Supplementary Materials Figures S2 and S3.

In the case of pDNA-based vectors, the percentage of positive cells (Figure 5A) was over 90% at 37 °C, but it decreased at 4 °C with SLN1 formulations. The intensity of fluorescence (Figure 5B) at 37 °C was also similar with all formulations. At 4 °C, the intensity also decreased for SLN1-based vectors, whereas it was similar or even higher for pDNA-DX-SLN2 and pDNA-HA-SLN2, respectively (Figure 5B).

In the case of mRNA-based vectors, the percentage of positive cells (Figure 5C) at 37 °C was over 92% for mRNA-DX-SLN1, mRNA-DX-SLN2, mRNA-HA-SLN1 and mRNA-HA-SLN2, whereas it ranged from 70% to 88% for the formulations prepared without polysaccharide. The percentage of entry was similar at 4 °C, but the intensity of fluorescence increased for SLN1-based formulations (Figure 5D).

#### 2.4.4. Cellular Uptake of the Vectors in HEK-293 Cells

**Figure 6:**
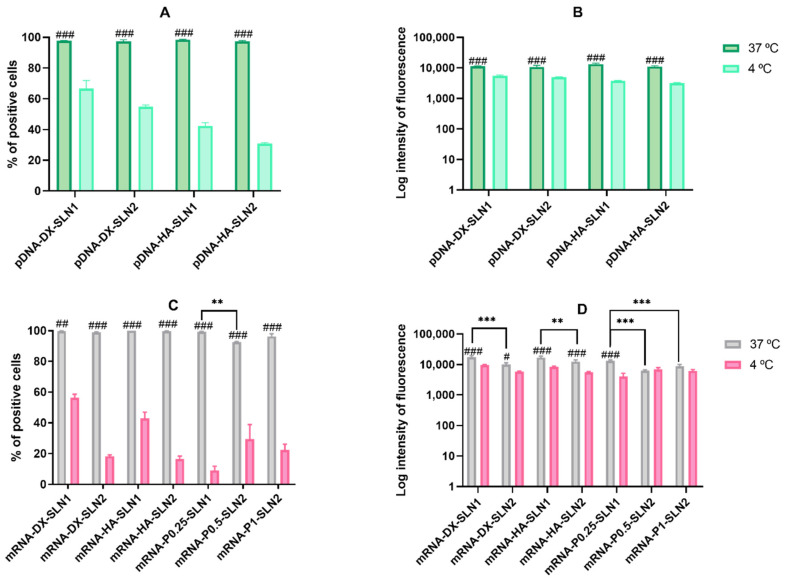
Flow cytometry analysis of cellular uptake of vectors, using Nile Red–labeled pDNA- and mRNA-SLNs in HEK-293 cells, at 37 and 4 °C. Data are expressed as mean ± standard deviation; *n* = 3 (**A**) Percentage of Nile Red positive HEK-293 cells 2 h after the treatment with pDNA vectors. (**B**) Intensity of fluorescence of Nile Red positive HEK-293 cells 2 h after the treatment with pDNA vectors. (**C**) Percentage of Nile Red positive HEK-293 cells2 h after the treatment with mRNA vectors. (**D**) Intensity of fluorescence of Nile Red positive HEK-293 cells 2 h after the treatment with mRNA vectors. ^#^ *p* < 0.05 with respect to the same vector at 4 °C. ^##^ *p* < 0.01 with respect to the same vector at 4 °C. ^###^ *p* < 0.001 with respect to the same vector at 4 °C. ** *p* < 0.01 with respect to the other formulation. *** *p* < 0.001 with respect to the other formulation.

Figure 6 shows the cell uptake and intensity of fluorescence of pDNA- and mRNA-based vectors labeled with Nile Red. HEK-293 cells were treated with the vectors at both 37 and 4 °C, and cell uptake was measured 2 h later. The histograms showing the cellular uptake are included in Supplementary Materials Figures S4 and S5.

In the case of pDNA-based vectors (Figure 6A), the percentage of positive cells was near 100% at 37 °C, whereas, at 4 °C, the entry decreased drastically by more than a half with HA containing vectors, and around a 50% with DX containing vectors. This reduction was also observed in terms of intensity (Figure 6B).

The entry of mRNA-based vectors at 37 °C (Figure 6C) was also close to 100% with all formulations, and, as in the case of pDNA-based vectors, the entry at 4 °C decreased extremely. The intensity of fluorescence (Figure 6D) was higher with SLN1 formulations than with SLN2 at 37 °C, and a drastic decrease on the intensity was observed at 4 °C, except for mRNA-P0.5-SLN2 and mRNA-P1-SLN2.

#### 2.4.5. Intracellular Disposition of Non-Viral Vectors

**Figure 7:**
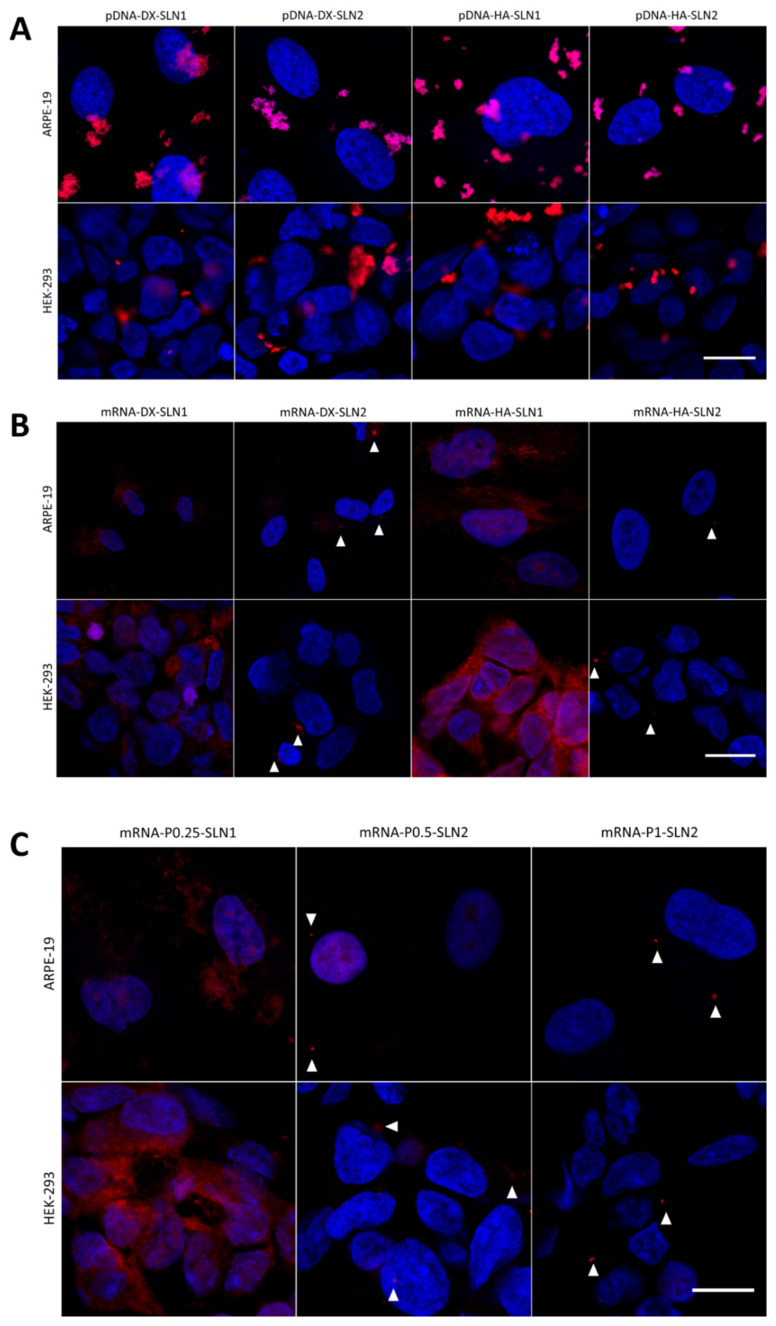
Fluorescence microscopy images 4 h after the addition of SLN1- and SLN2-based vectors in ARPE-19 and HEK-293 cells. (**A**) pcDNA3-EGFP plasmid labeled with Label IT^®^ Cy^®^5 vectors. (**B**) CleanCap™ Cyanine 5 EGFP mRNA (5moU) vectors formulated with P, DX and HA. (**C**) CleanCap™ Cyanine 5 EGFP mRNA (5moU) vectors formulated with P. Nuclei were labeled with DAPI (blue). Magnification 60×. Scale bar: 15 μm. White triangles indicate the mRNA.

Figure 7 shows the intracellular disposition of pDNA and mRNA in ARPE-19 and HEK-293 cells. Differences in the nucleic acid disposition, depending on the formulation and on the cell line, were observed.

In ARPE-19 cells, the disposition of pDNA was similar with all formulations. However, in HEK-293 cells, pDNA dots were smaller with the vectors DX-SLN1 and HA-SLN2.

In both cell lines, mRNA appeared less dispersed along the cytoplasm, when formulated in the vectors prepared with SLN1. This behavior is more noticeable in HEK-293 than in ARPE-19 cells.

### 2.5. Long-Term Stability Study of pDNA- and mRNA-Based Vectors

#### 2.5.1. Size, Polydispersity Index and ζ-Potential

**Figure 8:**
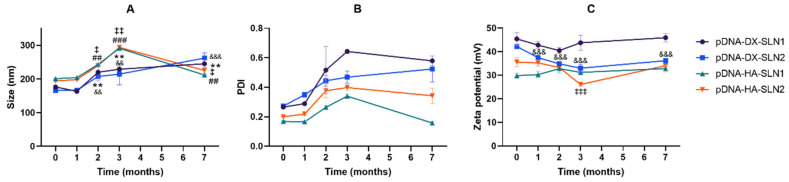
Size (**A**), polydispersity index (**B**) and ζ-potential (**C**) of pDNA-based vectors formulated with SLN1 and SLN2 at time zero and one, two and three months. Data are expressed as mean ± standard deviation; *n* = 3. ** *p* < 0.01 with respect to mRNA-DX-SLN1 at time zero. ^&&^ *p* < 0.01, ^&&&^ *p* < 0.001 with respect to pDNA-DX-SLN2 at time zero. ^##^ *p* < 0.01, ^###^ *p* < 0.001 with respect to pDNA-HA-SLN1 at time zero. ^‡^ *p* < 0.05, ^‡‡^ *p* < 0.01, ^‡‡‡^ *p* < 0.001 with respect to pDNA-HA-SLN2 at time zero.

**Figure 9:**
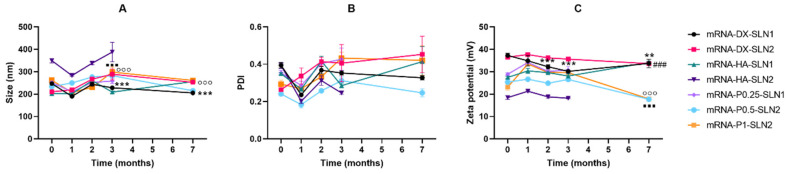
Size (**A**), polydispersity index (**B**) and ζ-potential (**C**) of mRNA-based vectors formulated with SLN1 and SLN2 at time zero and one, two and three months. Data are expressed as mean ± standard deviation; *n* = 3. ** *p* < 0.01, *** *p* < 0.001 with respect to mRNA-DX-SLN1 at time zero. ^###^ *p* < 0.001 with respect to mRNA-HA-SLN1 at time zero. °°° *p* < 0.001 with respect to mRNA-P0.5-SLN2 at time zero. ▪▪▪ *p* < 0.001 with respect to mRNA-P1-SLN2 at time zero.

Figure 8 and Figure 9 present the characterization data of pDNA- and mRNA-based vectors, respectively, over one, two, three and seven months. The particle size and PDI of pDNA formulations increased after two months of storage, at 4 °C (Figure 8A,B); the change of the size was higher for the vectors containing HA. After two months, the PDI of pDNA-DX-SLN1 and pDNA-DX-SLN2 increased above 0.4. The ζ-potential of SLN1 vectors was maintained during the seven months, but it decreased in the case of SLN2 formulations (Figure 8C).

In the case of mRNA formulations, after seven months, the mean size (Figure 9A) changed significantly for mRNA-P0.5-SLN2 and mRNA-DX-SLN1; it decreased 20 and 40 nm, respectively. PDI values were below 0.4 during the stability study (Figure 9B). Differences were observed after two months in ζ-potential for mRNA-DX-SLN1, and at month seven for all formulations except for mRNA-DX-SLN2 (Figure 9C).

#### 2.5.2. Binding, Protection and Release of Nucleic Acids

**Figure 10:**
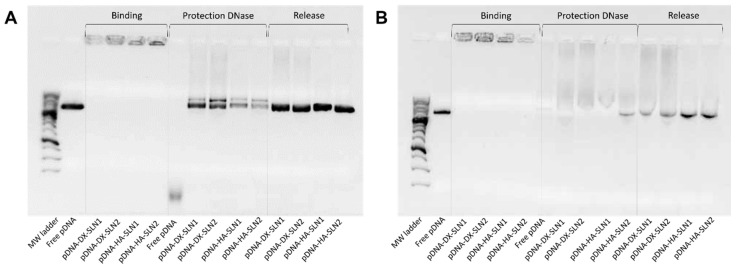
Long-term stability study of the binding, protection and release of pDNA-based vectors formulated with SLN1 and SLN2. (**A**) Binding, protection and release after one month of storage. (**B**) Binding, protection and release after three months of storage.

Gel electrophoresis of pDNA-based vectors were carried out at time zero (freshly prepared; Figure 2A), and after one, two and three months of storage at 4 °C (gel electrophoresis at month two is included in Supplementary Materials Figure S6). Figure 10 shows the gel electrophoresis at one month (Figure 10A) and three months (Figure 10B). Regarding the binding, the presence of bands on the loading wells and their absence on the corresponding lanes at both, one and three months, indicates that vectors maintained the pDNA binding capacity. In comparison to time zero, pDNA-HA vectors showed two bands instead one and less intense, indicating that these vectors maintained the ability to protect the pDNA only partially. The protection ability of all vectors decreased at two months of storage (Supplementary Materials Figure S6), and after three months, all vectors lost the pDNA protection capacity, as the pDNA fragmentation indicates. One month after the preparation, the vectors were able to release the nucleic acid.

**Figure 11:**
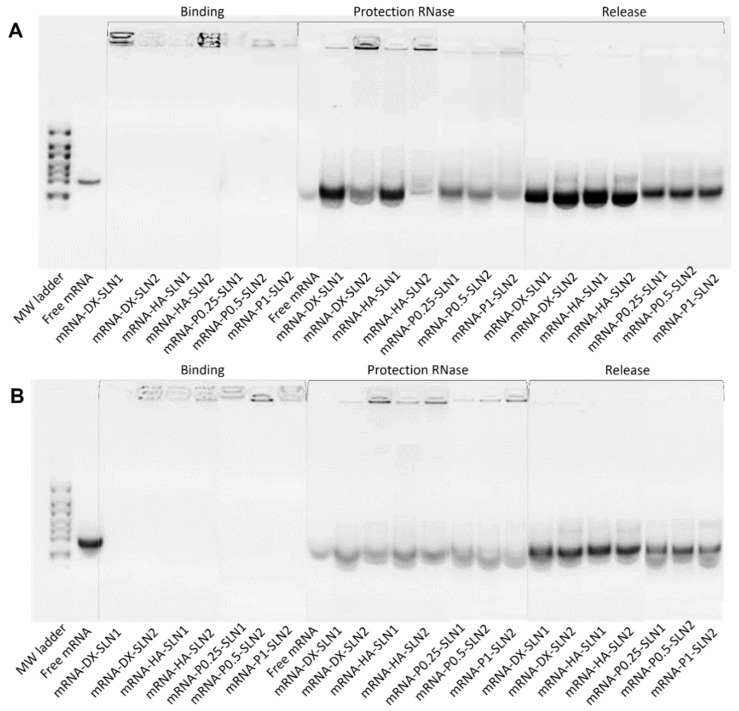
Long-term stability study of the binding, protection and release of mRNA-based vectors formulated with SLN1 and SLN2. (**A**) Binding, protection and release after one month of storage. (**B**) Binding, protection and release after three months of storage.

Gel electrophoresis of mRNA-based vectors were carried out at time zero (freshly prepared, Figure 2B), and after one, two and three months of storage at 4 °C (gel electrophoresis of two months is included in Supplementary Materials Figure S7). Figure 11 shows gel electrophoresis at month one (Figure 11A) and month three (Figure 11B). After one and three months of storage, the vectors maintained their ability to completely bind the mRNA. One month after the preparation, the vectors were still able to protect the mRNA, but from two months (Supplementary Materials Figure S7), the nucleic acid was much less protected, regardless of the formulation.

#### 2.5.3. Transfection Efficacy and Cell Viability

**Figure 12:**
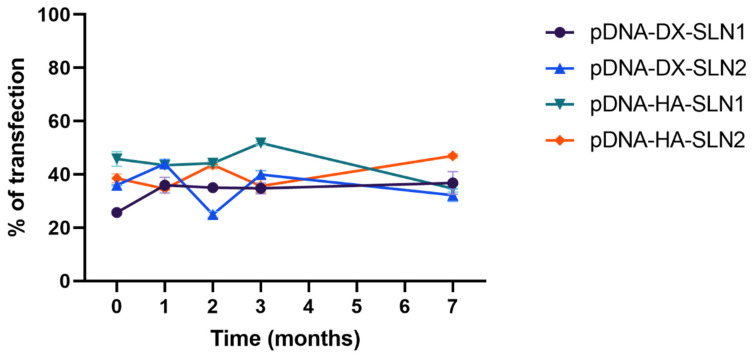
Percentage of ARPE-19 transfected cells after treatment with pDNA vectors after different times of storage. Data are expressed as mean ± standard deviation; *n* = 3.

Figure 12 shows the percentage of ARPE-19 cells transfected with pDNA-based vectors, at time zero (freshly prepared) and one, two, three and seven months after preparation. The percentage of transfected cells with pDNA-vectors remained stable during the seven-month evaluated.

**Figure 13:**
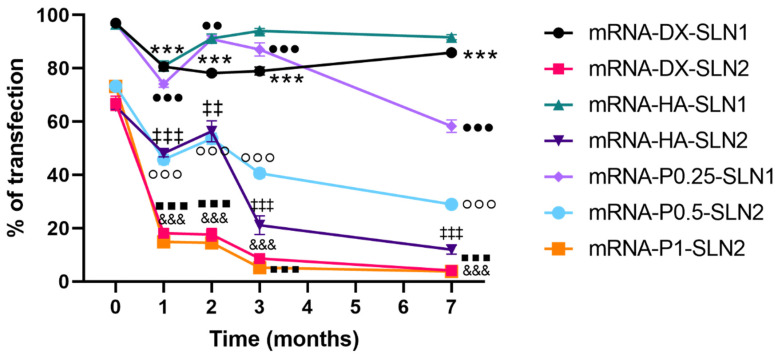
Percentage of ARPE-19 transfected cells after treatment with mRNA vectors after different times of storage. Data are expressed as mean ± standard deviation; *n* = 3. *** *p* < 0.001 with respect to mRNA-DX-SLN1 at time zero. ^&&&^ *p* < 0.001 with respect to mRNA-DX-SLN2 at time zero. ^‡‡^ *p* < 0.01, ^‡‡‡^ *p* < 0.001 with respect to mRNA-HA-SLN2 at time zero. ^●●^ *p* < 0.01 and ^●●●^ *p* < 0.001 with respect to mRNA-P0.25-SLN1 at time zero. °°° *p* < 0.001 with respect to mRNA-P0.5-SLN2 at time zero. ▪▪▪ *p* < 0.001 with respect to mRNA-P1-SLN2 at time zero.

In the case of mRNA (Figure 13) the transfection efficacy of mRNA-SLN2 vectors decreased significantly during the first three months of storage. The percentage of transfected cells felt below 25% at month one in the case of mRNA-DX-SLN2 and mRNA-P1-SLN2. When the vectors were prepared with SLN1, the percentage of transfection remained over 80% for three months for mRNA-P0.25-SLN1, and during the seven-month evaluated for mRNA-DX-SLN1 and mRNA-HA-SLN1.

There were no differences in cell viability values during the stability study for pDNA and mRNA formulations.

## 3. Discussion

The final formulations were prepared by combining the following components: SLNs, the nucleic acid (pDNA or mRNA), the cationic peptide protamine (P), and a polysaccharide, DX or HA. In our study, only the SLNs prepared with DOTAP as the only cationic lipid (SLN1) and those containing a mixture of DOTAP and DODAP (SLN2) showed physicochemical features adequate to be used as nucleic acid delivery systems.

The electrostatic interactions between the different components play a major role in the vector formation, conditioning the final structure. TEM photographs of mRNA-vectors (Figure 1) show an external layer or a corona surrounding the surface only when the polysaccharide is included in the final formulation. However, when mRNA was only complexed with P and SLN2, a characteristic multilamellar internal structure can be observed; this structure has been also documented when the mRNA is combined with P and DOTAP as cationic lipid [23].

To prepare the nanovectors, the nucleic acid was first condensed with P, which contributes to bind and protect the genetic material at intra and extracellular level (Supplementary Materials Figure S8). Regarding DX and HA, both polysaccharides possess suitable properties to improve nucleic acid delivery. The final pDNA formulations presented a particle size and a PDI similar to the plain SLNs but lower ζ-potential. Nonetheless, mRNA-based formulations showed higher particle size and PDI values, and lower superficial charge than those prepared with pDNA. Electrophoresis on agarose gels showed that pDNA was well protected, regardless of the formulation; however, the incorporation of DODAP to the SLNs resulted in a lower capacity to protect the mRNA, which indicates that the mRNA is more exposed to external agents, such as RNAses. The difference in the capacity of the vectors to protect the nucleic acid may be related to the unique structure of mRNA, which is a single-stranded molecule that folds into complex secondary and tertiary structures, and takes forms that differ from the double stranded pDNA [24].

We have evaluated the different vectors in two cell models, ARPE-19 and HEK-293 cells. These cell lines have been previously selected to study the behavior of SLN-based vectors as pDNA delivery systems, because of their different features in terms of cell division rate (which is lower in ARPE-19 cells) and of the main endocytic processes [25]. As can be seen in Figure 3 and Figure 4, all formulations were able to transfect both cell lines, regardless of the type of SLN, although the transfection efficacy varied depending on the cell line, on the type of nucleic acid and on the composition of the vectors. The efficacy of a nucleic acid delivery system depends on its ability to behave as a stable complex that provides protection of the genetic material against degradation, but is able to disassemble intracellularly to release it; both properties are conditioned by the physicochemical characteristics of the nanosystem. In this sense, the SLNs resulted necessary for transfection, and when cells were treated with a complex prepared with mRNA or pDNA, P, and DX or HA, the percentage of transfected cells was lower than 0.8% (data not shown).

In ARPE-19 cells at 37 °C, pDNA-based vectors (Figure 3A) induced a lower percentage of transfected cells than those containing mRNA (Figure 3C), but pDNA-mediated transfected cells provided higher fluorescence intensities; namely, pDNA transfection is more effective for protein production in this cell line. In HEK-293, pDNA-based vectors (Figure 4) induced lower transfection efficacy than mRNA-vectors in terms of transfected cells and protein production. Overall, cellular uptake of all vectors, pDNA and mRNA, was over 90%; therefore, the bottleneck for a successful pDNA transfection seems to be the nuclear entry, despite our systems contains P, which favors the transcription process and the entry of the pDNA into the nucleus, thanks to their nuclear localization signals [26]. Unfortunately, an effective nuclear transport system has yet to be stablished [27]. Among pDNA vectors, the highest transfection efficacy was observed with HA-containing vectors in both cell lines, and in ARPE-19 especially with the vector pDNA-HA-SLN1. Regarding mRNA formulations, the inclusion of a polysaccharide did not have a great impact on the in vitro transfection efficacy.

Designing nanosystems to modulate intracellular nucleic acid release and stability is a key challenge to broad the therapeutic potential of nucleic acid based medicinal products. All mRNA formulations showed the highest transfection efficacy at 24–48 h, and the protein expression decreased notably at 96 h, whereas pDNA formulations showed the maximum transfection at 72–96 h; in both cases, it lasted at least 11 days (Supplementary Materials Figure S9). The synthesis of the encoded protein with mRNA is faster and its expression is temporary, which makes it a more predictable molecule than pDNA; however, mRNA provides short-term transfection. Most mRNA applications are focusing on immunotherapy, and particularly on cancer, due to the immunostimulatory capacity of mRNA together with the transient nature of the encoded antigen and the versatility of applications, including prophylaxis, therapy and personalized vaccines [28,29]. Transient expression could be also beneficial for introducing antiapoptotic factors or genome editing enzymes [27]. However, in other cases, long-term expression is required, such as in protein replacement therapy intended for the supplementation of infra-expressed and not functional proteins, or for the expression of foreign proteins [6]. Therefore, the duration of transfection should be considered and not only the rate of transfected cells and the efficiency of the protein production. In our study, although mRNA vectors were able to transfect a larger number of cells, pDNA-mediated transfection in ARPE-19 cells was the most effective in terms of the amount of protein synthetized per cell. The production of a large amount of protein, even with a low percentage of transfected cells, may be a desired objective in gene augmentation to produce a protein in a cell to be secreted and reach the target tissue.

All the components that form the vector determine the interaction with the target cells, and as a consequence, the internalization process, the intracellular behavior of the genetic material and the transfection capacity [30,31]. It is well-known that the degree of cellular uptake and endosomal escape of the vectors condition transfection efficiency [32]. The major entry mechanism of SLN-based vectors is the endocytosis. The two main endocytic processes are reported to be pinocytosis and phagocytosis. The former is mainly associated to nanoparticle uptake, and different pathways are involved (micropinocytosis, clathrin-mediated and caveolin dependent or independent) [33]. The predominant entry pathway depends on the target cells and on the composition and physicochemical characteristics of the non-viral vectors [14]. The endocytosis is an energy-dependent and temperature-dependent process, and it is inhibited at 4 °C, because cells consume less ATP and block the active transport at this temperature [34,35].

In ARPE-19 cells, the percentage of transfected cells decreased significantly at 4 °C for almost all formulations, and mainly for pDNA formulations and mRNA-DX-SLN2. Surprisingly, the fluorescence intensity of the vectors containing DODAP (SLN2) did not change or even increased at low temperature. The lower cellular uptake of pDNA-SLN1 formulations at 4 °C explains the decrease of the transfection efficacy of these formulations. On the contrary, the percentage of entry of pDNA-SLN2 and mRNA vectors was hardly affected at 4 °C, which reveals the presence of energy-independent mechanisms; actually, lipid exchange might occur at 4 °C and contribute to the intracellular transport of the vectors [34]. It has to be taken also in mind that, due to the nature of biological systems, several dynamic processes might take place in parallel, which might turn in compete with one another [36]. The higher protein production observed at 4 °C, despite the lower number of transfected cells, indicates that in ARPE-19 cells energy-independent mechanisms are more effective to induce protein production, principally in the case of the vectors containing DODAP.

In HEK-293 cells, cell uptake and fluorescence intensity of transfection decreased drastically at 4 °C, but surprisingly, the percentage of transfected cells by the pDNA vectors was not affected. This cell line presents a high caveolae-dependent endocytic activity, and the blocking of the active transport has a very relevant influence on the uptake. In the case of mRNA vectors, the efficacy of transfection of mRNA-SLN1 vectors was less affected than the SLN2 formulations at low temperature. Taking in mind all of these results, we can conclude that, in HEK-293, cell energy-dependent entry mechanisms are the most effective for protein production, regardless of the kind of the SLN used for preparing the pDNA vectors, and mainly for SLN2 formulations in the case of mRNA. Therefore, protein production is favored by energy-dependent or -independent mechanisms, depending on the cell line, SLN composition and the nucleic acid delivered, pDNA vs. mRNA. Endosomal escape is recognized as the rate-limiting step for mRNA delivery [37], and ionizable lipids, such as DODAP, could facilitate this process. Nevertheless, recently, Patel et al. have reported that late endosome/lysosome formation is essential for the functional delivery of exogenously presented mRNA [38]. The balance between endosomal escaping capability and stability of translocated nucleic acids in cytoplasm is essential for an effective transfection. This balance may be modulated by an appropriate delivery system. If mRNA is easily transported and delivered into the cytoplasm, its poor stability will make that the transfection results in a short pattern. However, if the delivery system slows down the endosomal escape and provides long-protection of mRNA, a sustained expression may be achieved. In the case of pDNA, which shows higher stability than mRNA, the limiting step in transfection would be more related to the endosomal escape than to the cytoplasmic stability [39]. It is essential that the early steps of the development of nucleic acid medicinal products include mechanistic studies in the target cell for an in depth understanding of the intracellular nucleic acid nanomedicines’ behavior. This knowledge will allow a properly design of the formulations specifically adapted to the nucleic acid features, clinical application and therapeutic purpose.

In the present work, the type of formulation had a greater influence on the intracellular disposition of mRNA than that of pDNA (Figure 7). Overall, mRNA appeared less dispersed along the cytoplasm with the vectors prepared only with DOTAP (SLN1). On the agarose gel (Figure 2), vectors prepared with both SLN1 and SLN2 were able to completely bind the mRNA; however, the formulations prepared with DOTAP and DODAP (SLN2) showed a lower protection degree, which is usually associated to a less condensation capacity. The differences in the intracellular disposition were related not only to the capacity of the vectors to bind, condense and protect the nucleic acid, but also to the entry mechanism and the intracellular trafficking of the vectors, which are cell-line-dependent processes. Unlike what happens in ARPE-19 cells, in HEK-293 cells, mRNA appeared more dispersed in the cytoplasm, which may be indicative of a higher exposure to degradation. Consequently, mRNA was most effective in ARPE-19 than in HEK-293 cells, even if a lower dose of mRNA was administered in retinal cells.

Efficacy and stability of the delivery systems should be guaranteed during storage over time. In fact, thermal stability of nucleic acid-based medicinal products is a major issue, especially relevant in the case of vaccines, since storage conditions involve a logistical problem for stockpiling and distribution, particularly in countries that lack infrastructure to maintain the cold chain [40]. We have evaluated the physicochemical characteristics and the transfection efficacy of our vectors along seven months of storage at 4 °C. The changes detected in size, PDI, zeta potential and in the agarose gel studies did not always correlate with the transfection efficacy. The pDNA formulations showed physicochemical changes from the second month, but transfection efficacy was maintained for seven months in vitro. On the contrary, mRNA vectors were more stable in terms of size, zeta potential and in the agarose gel studies, but transfection decreased drastically from the first month with the vectors containing the SLNs prepared with DOTAP and DODAP (SLN2). The vectors prepared with mRNA and the SLNs containing only DOTAP showed a percentage of transfection higher than 80% during the first three months of storage, and it lasted for seven months, except for the formulation prepared without polysaccharide. Therefore, in the formulations prepared with SLN containing only DOTAP as cationic lipid (SLN1), the inclusion of a polysaccharide confers stability; in addition, it provides other beneficial properties such as stealth capacity and ability to modulate the mechanism of entry to the target cell [15,25,30]. The lower number of positive charges of the lipid DODAP is related to its lower capacity to condense the genetic material, which could be the reason of the worse stability of SLN2-based vectors, especially for mRNA. Considering not only the efficacy but also the long-term stability, vectors prepared only with the cationic lipid DOTAP seem to be the most promising formulations.

## 4. Materials and Methods

### 4.1. Materials

Precirol^®^ ATO 5 (glyceryl palmitostearate) was generously provided by Gattefossé (Madrid, Spain); 1,2-dioleoyl-3-trimethylammonium-propane chloride salt (DOTAP) and 1,2-dioleoyl-3-dimethylammonium-propane (DODAP) were obtained from Avanti Polar-lipids, Inc. (Alabaster, AL, USA); and Tween 80 and dichloromethane were obtained from Panreac (Madrid, Spain). Protamine sulfate salt Grade X (P), dextran (Mw of 3.26 KDa) (DX) and Nile Red were purchased from Sigma-Aldrich (Madrid, Spain). Hyaluronic acid (Mw of 100 KDa) (HA) was acquired from Lifecore Biomedical (Chaska, MN, USA).

The plasmid pcDNA3-EGFP (6.1 kb) was kindly provided by the laboratory of Professor BHF Weber (University of Regensburg, Regensburg, Germany), and CleanCap™ EGFP mRNA (5moU) and CleanCap™ Cyanine 5 EGFP mRNA (5moU) were purchased from TriLink BioTechnologies. All of the nucleic acids encoded green fluorescent protein (GFP). A Label IT^®^ Cy^®^5 Nucleic Acid Labeling Kit was obtained from Mirus.

The materials employed for the electrophoresis on agarose gel were acquired from Bio-Rad (Madrid, Spain). Deoxyribonuclease I (DNase I) and sodium dodecyl sulfate (SDS) were obtained from Sigma-Aldrich, GelRed™ from Biotium (Fremont, CA, USA) and Ambion™ RNase I from Life Technologies (ThermoFisher Scientific, Madrid, Spain).

Human Retinal Pigmented Epithelium (ARPE-19) and Human Embryonic Kidney (HEK-293) cells were purchased from American Type Culture Collection (ATCC, Manassas, VA, USA). Cell culture reagents, including Dulbecco’s Modified Eagle’s Medium/Nutrient Mixture F-12 (DMEM/F-12), fetal bovine serum, penicillin–streptomycin and trypsin-EDTA, were acquired from Life Technologies (ThermoFisher Scientific, Madrid, Spain). Eagle’s Minimum Essential Medium (EMEM) was purchased from LGC Promochem (Barcelona, Spain).

Reporter lysis buffer was provided by Promega Biotech Ibérica (Madrid, Spain), and 4′,6-diamidine-2′-phenylindole dihydrochloride (DAPI)-fluoromount-G by Southern Biotech (Birmingham, AL, USA). Paraformaldehyde (PFA) was obtained from Panreac, while phosphate buffered saline (PBS) and HEPES buffer were purchased from Gibco (ThermoFisher Scientific, Madrid, Spain). Lipofectamine™ 2000 Lipid-Reagent was acquired from Life Technologies (ThermoFisher Scientific, Madrid, Spain) and Cell Couniting Kit-8 (CCK-8) from Sigma-Aldrich (Madrid, Spain). The 7-Amino-Actinomycin D (7-AAD) Viability Dye was provided by Beckman Coulter (Brea, CA, USA).

### 4.2. Preparation of SLNs and Vectors

Four different SLNs, SLN1, SLN2, SLN3 and SLN4, were prepared by the solvent emulsification-evaporation method previously described [41].

Table 3 shows the composition of the SLNs. SLN1 was obtained by the sonication of the oil phase containing Precirol^®^ ATO 5 and dichloromethane in an aqueous phase of the cationic lipid DOTAP (0.4%, *w/v*) and Tween 80 (0.1%, *w/v*), whereas SLN2 contained a mixture of DOTAP (0.2% *w/v*) and DODAP (0.2% *w/v*) as cationic and ionizable lipids, respectively. SLN3 was prepared with the ionizable lipid DOBAQ (0.4%, *w/v*), and SLN4 with a mixture of DOTAP (0.2% *w/v*) and DOBAQ (0.2% *w/v*).

In order to elaborate SLN-based vectors, the nucleic acid (pcDNA3-EGFP plasmid, CleanCap™ EGFP mRNA (5moU)) was first mixed for 5 min with the protamine (P) solubilized in water. Then, when corresponding, an aqueous solution of a polysaccharide, dextran (DX) or hyaluronic acid (HA) was added to the previous mixture, and they were kept in contact for 15 min. Finally, the suspension of SLNs was added to the complexes. The final vectors were obtained by the electrostatic interactions between all the components. The weight ratios of the components are summarized in Table 4.

### 4.3. Size, PDI and ζ-Potential Measurement

Size and polydispersity index (PDI) of SLNs and final vectors were measured by photon correlation spectroscopy, and ζ potential by laser doppler velocimetry, in a ζsizer Nano series-Nano ZS (Malvern Instruments, Worcestershire, UK). All samples were dilute in Milli-Q™ water (EDM Millipore, Billerica, MA, USA).

### 4.4. Transmission Electron Microscopy (TEM) Images

SLN1, SLN2, SLN1-based and SLN2-based vectors were observed by Transmission Electronic Microscopy (TEM), using electron microscopy negative staining. To this end, 10 μL of the sample was adsorbed for 60 s onto glow discharged carbon coated grids. Then, the remaining liquid was removed, via blotting on filter paper, and the staining was carried out with 2% uranyl acetate for 60 s.

Visualization of samples was performed in a Philips EM208S TEM. For acquisition of digital images, an Olympus SIS purple digital camera was used. Technical and human support for TEM was provided by the Advanced Research Facilities (SGIker) of Analytical and High Resolution Microscopy in Biomedicine at the University of the Basque Country UPV/EHU (Leioa, Basque Country, Spain).

### 4.5. Agarose Gel Electrophoresis Assay

The studies of the pDNA binding capacity, the protection from DNase I and the release from the vectors were performed in 0.7% agarose gel electrophoresis labeled with Gel Red™. The gels were run for 30 min at 120 V, and immediately they were analyzed with the Uvitec Uvidoc D-55-LCD-20 M Auto transilluminator. To evaluate binding capacity, vectors were diluted in MilliQ™ water to a final concentration of 0.03 μg pDNA/μL in the gel. The protection was analyzed by the addition of 1 U DNase I/2.5 μg pDNA; mixtures were incubated at 37 °C for 30 min in a heater. Then, the samples were removed from the heater and mixed with a SDS solution (final concentration of 1%), at room temperature, to release the nucleic acid. The same SDS solution was added to the vectors to unbind the plasmid in the release studies. Two controls for the integrity of the pDNA were included in the gels: 1 kb pDNA ladder from NIPPON Genetics Europe (Dueren, Germany) and untreated pcDNA3-EGFP plasmid.

In order to study the mRNA binding capacity, the protection from RNase I digestion and the release from the vectors 1.2% agarose gel electrophoresis for 60 min at 75 V containing Gel Red™ was used. The bands were analyzed with Uvitec Uvidoc D-55-LCD-20 M Auto transilluminator (Cambridge, UK). To evaluate binding capacity, vectors were diluted in MilliQ™ water to a final concentration of 0.12 μg/μL mRNA in the gel. The protection was analyzed by the addition of 6 U RNase I/μg mRNA; mixtures were incubated at 37 °C, for 40 min, in a heater. Then, the samples were removed from the heater and mixed with an SDS solution (final concentration of 1%), at room temperature, to release the nucleic acid. The same SDS solution was added to the vectors, to unbind the plasmid in the release studies. RiboRuler High Range RNA Ladder and untreated CleanCap™ EGFP mRNA (5moU) as controls were included as controls in the gels, to compare the integrity of the mRNA.

### 4.6. Cell Culture Studies

In vitro cell studies were carried out in two cell lines: Human Retinal Pigmented Epithelial (ARPE-19) and Human Embryonic Kidney (HEK-293) cells. ARPE-19 were maintained in culture in Dulbecco’s Modified Eagle Medium:Nutrient Mixture F-12 (DMEM/F-12), and HEK-293 cells were cultured in Eagle’s Minimum Essential Medium (EMEM). Both mediums were supplemented with 10% fetal bovine serum and 1% penicillin and streptomycin antibiotic. Cells cultures were incubated at 37 °C, in a 5% CO_2_ air atmosphere, changing the medium every 2 or 3 days; they were subcultured every 7 days.

#### 4.6.1. Transfection Efficacy and Cell Viability

The pDNA and mRNA vectors were prepared 24 and 72 h before their addition, respectively, and maintained at 4 °C before their use. ARPE-19 cells were cultured at a density of 60,000 cells per well, in 12-well plates, 72 h before the addition of the vectors, in order to ensure the formation of a cell monolayer. HEK-293 cells were cultured at a density of 150,000 cells per well, in 24-well plates, 24 h before the addition of the vectors, when a 75–85% confluence was reached. Then, part of the medium was removed, leaving the enough volume to cover the cells, and a total volume of 75 μL of each vector diluted in HBS (equivalent to 2.5 μg or 1.5 μg of pDNA or mRNA, respectively) was added to each well. The dose of mRNA to be added to the cells was optimized in order to better detect differences in the transfection efficacy (Supplementary Materials Figure S10). After incubation for 4 h, at 37 °C, in a 5% CO_2_ air atmosphere, vectors were removed, and cells were refreshed with 1 mL of complete medium. Cells were kept growing during 72 h in the pDNA experiments, and during 48 h in the mRNA experiments.

In order to analyze transfection efficacy and cell viability, the cells were washed with 500 μL of PBS and then detached by incubation with 500 μL of trypsin-EDTA for 10 min. Thereafter, the cell suspension was centrifuged at 1000 rpm for 5 min. The supernatant was discarded, and the pellet of cells was resuspended in 500 μL of PBS.

In both cell lines, the percentage of transfected cells and intensity of fluorescence, indicative of the amount of EGFP produced, and the cell viability were measured by using a CytoFLEX flow cytometer (Beckman Coulter). For each sample, 10,000 events were collected. Transfection efficacy was measured at 525 nm (FITC), and cell viability was determined at 610 nm (ECD), after the addition of 7-Amino-Actinomycin D (7-AAD) Viability Dye to the samples. Moreover, 7-AAD labels fluorescently dead cells.

The effect of temperature on cell transfection was studied by incubation of ARPE-19 and HEK-293 cells at 4 °C, for 30 min, prior to the addition of the vectors. The cells were maintained for 4 h, at 4 °C, and then cells were treated as explained above.

#### 4.6.2. Cellular Uptake

The internalization of the vectors was studied in ARPE-19 and HEK-293 cells, by using SLNs labeled with the fluorescent dye Nile Red (λ = 590 nm) to prepare the vectors, according to a previously reported method [42]. For this purpose, when preparing SLNs, Nile Red was dissolved in dichloromethane together with Precirol^®^ ATO 5.

Two hours after the addition of 75 μL of the vectors, the culture medium was retired and cells were detached from plates, as described in Section 4.6.1. for the cytometry analysis of transfected cells. The entrance of the vectors was analyzed by using a CytoFLEX flow cytometer (Beckman Coulter) at 610 nm (ECD). For each sample, 10,000 events were collected.

In addition, the effect of temperature on cell uptake was studied by incubation of ARPE-19 and HEK-293 cells at 4 °C for 30 min prior to the addition of the vectors. Once the Nile Red–labeled vectors were added to cell cultures, the cells were maintained at 4 °C for additional 2 h. Finally, the cells were collected to evaluate the vector uptake by flow cytometry, as described above.

#### 4.6.3. Intracellular Disposition

To evaluate the intracellular location of the vectors, 35,000 cells and 150,000 cells of ARPE-19 and HEK-293, respectively, were seeded in Millicell EZ slides (Millipore) with 1 mL per well at 37 °C and 5% CO_2_ air atmosphere 24 h before experiment.

For mRNA assays, 75 μL of vector equivalent to 0.8 μg of CleanCap™ Cyanine 5 EGFP mRNA (5moU) as nucleic acid was added to each well. For pDNA assays pcDNA3-EGFP plasmid was labeled with Label IT^®^ Cy^®^5, following manufacturer’s instructions, and 75 μL of vector equivalent to 2.5 μg of pcDNA3-EGFP plasmid was added to each well. After 4 h, the slides were washed with PBS and fixed with PFA 4%. DAPI-fluoromount-G™ was used as the mounting fluid, to label the nuclei. The slides were then were examined under a Zeiss LSM800 confocal microscope (Oberkochen, Germany).

### 4.7. Long-Term Stability Study of pDNA- and mRNA-Based Vectors

The pDNA- and mRNA-based vectors were stored at 4 °C during 7 months. Both, pDNA- and mRNA-based vectors were elaborated with 2.5 μg of nucleic acid. Vectors were characterized at different times (1, 2, 3 and 7 months), in terms of size, PDI and ζ potential, as described in Section 4.3, as well as the binding, protection and release capacity of the nucleic acids from the delivery system. The percentage of transfection was also quantified in ARPE-19 at the different storage times.

#### Statistical Analysis

Results are reported as mean values ± standard deviation (SD). Statistical analysis was performed by using IBM^®^ SPSS^®^ Statistics 25 (IBM, Armonk, NY, USA). The normal distribution of samples was assessed by the Shapiro–Wilk test, and homogeneity of variance by the Levene test. The different formulations were compared with ANOVA and Student’s *t*-test. Differences were considered statistically significant at *p* < 0.05.

## 5. Conclusions

The present work shows that formulation-related factors have a greater influence on nucleic acid delivery, transfection efficiency and long-term stability of mRNA vectors than pDNA vectors. The formulations prepared with DOTAP as a unique cationic lipid showed a better stability profile during storage than those also containing the ionizable lipid DODAP; moreover, the addition of a polysaccharide to DOTAP formulations improved the long-term stability.

The therapeutic potential of the nucleic-acid-based medicinal products designed is also highly conditioned by the intracellular behavior of the nucleic acid nanomedicines in the target cell. Protein production was conditioned by energy-dependent or independent entry mechanisms, depending on the cell line, SLN composition and kind of nucleic acid delivered. On the one hand, mRNA vectors were able to transfect a larger number of cells, and the protein expression was faster and less permanent than that obtained with pDNA vectors; therefore, these mRNA systems may be interesting for immunization or gene editing applications. On the other hand, pDNA formulations were the most effective in terms of production of encoded protein in retinal cells, which would be interesting for protein replacement therapy when the therapeutic objective is to produce a protein in a cell to be secreted and reach the target tissue.

## Figures and Tables

**Table 1:** Physical characterization of solid lipid nanoparticles (SLNs).

SLNs	Cationic/Ionizable Lipid	Size (nm)	PDI	ζ-Potential (mV)
SLN1	DOTAP/-	185.1 ± 3.5	0.30 ± 0.03	+59.5 ± 1.9 ^&^
SLN2	DOTAP/DODAP	208.8 ± 0.4	0.27 ± 0.01	+50.2 ± 1.1 ^&^
SLN3	-/DOBAQ	423.5 ± 51.7 ***	0.57 ± 0.05	−28.0 ± 0.8 ^&^
SLN4	DOTAP/DOBAQ	211.3 ± 3.5	0.36 ± 0.01	+42.4 ± 1.2 ^&^

*** *p* < 0.001 with respect to SLN1, SLN2 and SLN4. ^&^ *p* < 0.001 with respect to the all SLNs. SLN: solid lipid nanoparticle. PDI: polydispersity index. DOTAP: 1,2-dioleoyl-3-trimethylammonium-propane. DODAP: 1,2-dioleoyl-3-dimethylammonium-propane. DOBAQ: N-(4-carboxybenzyl)-N,N-dimethyl-2,3-bis(oleoyloxy)propan-1-aminium. Data are expressed as mean ± standard deviation; *n* = 3.

**Table 2:** Physical characterization of SLNs-based vectors.

Nucleic Acid	Vectors	Size (nm)	PDI	ζ-Potential (mV)
pcDNA3-EGFP plasmid	pDNA-DX-SLN1	176.4 ± 0.4	0.27 ± 0.01	+45.4 ± 2.7
	pDNA-DX-SLN2	165.8 ± 1.7	0.27 ± 0.01	+42.2 ± 0.9
	pDNA-DX-SLN4	211.9 ± 14.6 *	0.40 ± 0.07 ^###^	+32.6 ± 0.9
	pDNA-HA-SLN1	201.2 ± 1.3	0.17 ± 0.01	+29.8 ± 1.1 ^&&&^
	pDNA-HA-SLN2	194.2 ± 0.8	0.20 ± 0.00	+35.6 ± 1.9 ^&^
CleanCap™ EGFP mRNA (5moU)	mRNA-DX-SLN1	246.8 ± 1.3	0.39 ± 0.02	+37.2 ± 1.0
	mRNA-DX-SLN2	210.1 ± 0.8 **	0.26 ± 0.01	+36.5 ± 0.3
	mRNA-HA-SLN1	202.4 ± 2.2 **	0.35 ± 0.00	+27.5 ± 0.6
	mRNA-HA-SLN2	349.2 ± 9.9 ^●●●^	0.39 ± 0.02	+18.5 ± 0.9 ^●^
	mRNA-P0.25-SLN1	251.6 ± 6.5	0.35 ± 0.01	+28.8 ± 0.7
	mRNA-P0.5-SLN2	233.7 ± 2.8	0.24 ± 0.00	+25.4 ± 0.6
	mRNA-P1-SLN2	261.7 ± 4.0	0.29 ± 0.02	+23.1 ± 1.3

* p < 0.05 with respect to pDNA-DX-SLN2. ** *p* < 0.01 with respect to mRNA-DX-SLN1, mRNA-P0.25-SLN1, mRNA-P0.5-SLN2 and mRN-P1-SLN2. ^●●●^ *p* < 0.001 with respect to the rest of mRNA vectors. ^###^ *p* < 0.001 with respect to the rest of pDNA vectors. ^&^ *p* < 0.05 with respect to mRNA-HA-SLN1. ^&&&^ *p* < 0.001 with respect to pDNA-DX-SLN1 and pDNA-DX-SLN2. ^●^ *p* < 0.05 with respect to mRNA-DX-SLN1 and mRNA-DX-SLN2. PDI: polydispersity index. DX: dextran. HA: hyaluronic acid. P: protamine. SLN: solid lipid nanoparticle. Data are expressed as mean ± standard deviation; *n* = 3.

**Table 3:** Composition of SLNs.

Type of SLN	Cationic Lipid (%)			Tween 80 (%)
	DOTAP	DODAP	DOBAQ	
SLN1	0.4			0.1
SLN2	0.2	0.2		0.1
SLN3			0.4	0.1
SLN4	0.2		0.2	0.1

SLN: solid lipid nanoparticle. DOTAP: 1,2-dioleoyl-3-trimethylammonium-propane. DODAP: 1,2-dioleoyl-3-dimethylammonium-propane. DOBAQ: N-(4-carboxybenzyl)-N,N-dimethyl-2,3-bis(oleoyloxy)propan-1-aminium.

**Table 4:** Weight ratios of the vectors.

Name of the Vector	Nucleic Acid	Polysaccharide	SLN	Weight Ratio (w:w:w:w)
pDNA-DX-SLN1	pDNA	DX	SLN1	DX:P:DNA:SLN1 1:2:1:5
pDNA-DX-SLN2	pDNA	DX	SLN2	DX:P:DNA:SLN2 1:2:1:5
pDNA-DX-SLN4	pDNA	DX	SLN4	DX:P:DNA:SLN4 1:2:1:5
pDNA-HA-SLN1	pDNA	HA	SLN1	HA:P:DNA:SLN1 0.5:2:1:2
pDNA-HA-SLN2	pDNA	HA	SLN2	HA:P:DNA:SLN2 0.5:2:1:5
mRNA-DX-SLN1	mRNA	DX	SLN1	DX:P:mRNA:SLN1 1:0.25:1:5
mRNA-DX-SLN2	mRNA	DX	SLN2	DX:P:mRNA:SLN2 1:1:1:5
mRNA-HA-SLN1	mRNA	HA	SLN1	HA:P:mRNA:SLN1 0.5:0.5:1:5
mRNA-HA-SLN2	mRNA	HA	SLN2	HA:P:mRNA:SLN2 0.5:1:1:5
mRNA-P-SLN1	mRNA	-	SLN1	P:mRNA:SLN1 0.25:1:5
mRNA-P0.5-SLN2	mRNA	-	SLN2	P:mRNA:SLN2 0.5:1:5
mRNA-P1-SLN2	mRNA	-	SLN2	P:mRNA:SLN2 1:1:5

DX: dextran. HA: hyaluronic acid. P: protamine. SLN: solid lipid nanoparticle.

## Supplementary Materials

The following are available online. Figure S1: Binding, protection and release capacity of pDNA-DX-SLN4. Figure S2: Flow cytometry analysis of cellular uptake of pDNA-vectors, using Nile Red–labeled SLNs in ARPE-19. Figure S3: Flow cytometry analysis of cellular uptake of mRNA-vectors, using Nile Red–labeled SLNs in ARPE-19. Figure S4: Flow cytometry analysis of cellular uptake of pDNA-vectors, using Nile Red–labeled SLNs in HEK-293 cells. Figure S5: Flow cytometry analysis of cellular uptake of mRNA-vectors, using Nile Red–labeled SLNs in HEK-293 cells. Figure S6: Study of the binding, protection and release of pDNA-based vectors formulated with SLN1 and SLN2 after two months of storage. Figure S7: Study of the binding, protection and release of mRNA-based vectors formulated with SLN1 and SLN2 after two months of storage. Figure S8: Microscopy images of the intracellular disposition in ARPE-19 cells of mRNA combined with P, SLN1 and the combination of both components. Figure S9: Microscopy images of ARPE-19 cells 24 h, 48 h, 72 h, 96 h and 11 days after transfection with pDNA- and mRNA-SLN1 vectors. Figure S10: Optimization of the dose of mRNA-based vectors in ARPE-19 cells.
