# Supplementary material for: Nucleic Acid Delivery by Solid Lipid Nanoparticles Containing Switchable Lipids: Plasmid DNA vs. Messenger RNA
Source: Molecules. 2020 Dec 18;25(24):5995. doi: 10.3390/molecules25245995 (PMC7766580; doi:10.3390/molecules25245995)
Supplement: Supplementary file 1 [file molecules-25-05995-s001.pdf]

Supplementary Materials: Nucleic Acid Delivery by Solid Lipid Nanoparticles Containing Switchable Lipids: Plasmid DNA vs. Messenger RNA

Itziar Gómez-Aguado [1], Julen Rodríguez-Castejón [1], Mónica Vicente-Pascual [1], Alicia Rodríguez-Gascón [1,2], Ana del Pozo-Rodríguez [1,2,*] and María Ángeles Solinís Aspiazu [1,2,*]

[1] Pharmacokinetic, Nanotechnology and Gene Therapy Group (PharmaNanoGene), Faculty of Pharmacy, Centro de investigación Lascaray ikergunea, University of the Basque Country UPV/EHU, Paseo de la Universidad 7, 01006 Vitoria-Gasteiz, Spain; itziar.gomez@ehu.eus (I.G.-A.); julen.rodriguez@ehu.eus (J.R.-C.); monica.vicente@ehu.eus (M.V.-P.); alicia.rodriguez@ehu.eus (A.R.-G.);

[2] Bioaraba, Pharmacokinetic, Nanotechnology and Gene Therapy Group (PharmaNanoGene), 01006 Vitoria-Gasteiz, Spain

* Correspondence: ana.delpozo@ehu.eus (A.d.P.-R.); marian.solinis@ehu.eus (M.Á.S.A.); Tel.: +34-945014498 (A.d.P.-R.); +34-945013469 (M.Á.S.A.)

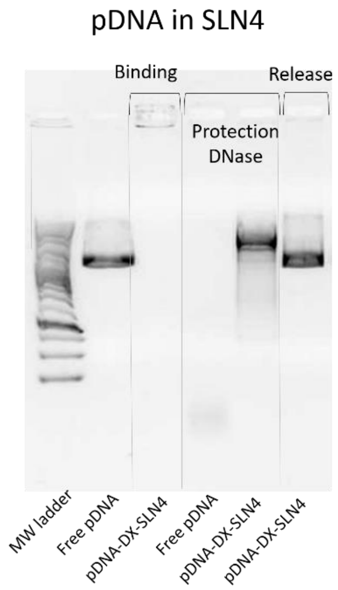

Figure S1. Binding, protection and release capacity of pDNA-DX-SLN4. Vector was able to bind and release the pDNA. However, vector showed low capacity of protection of pDNA.

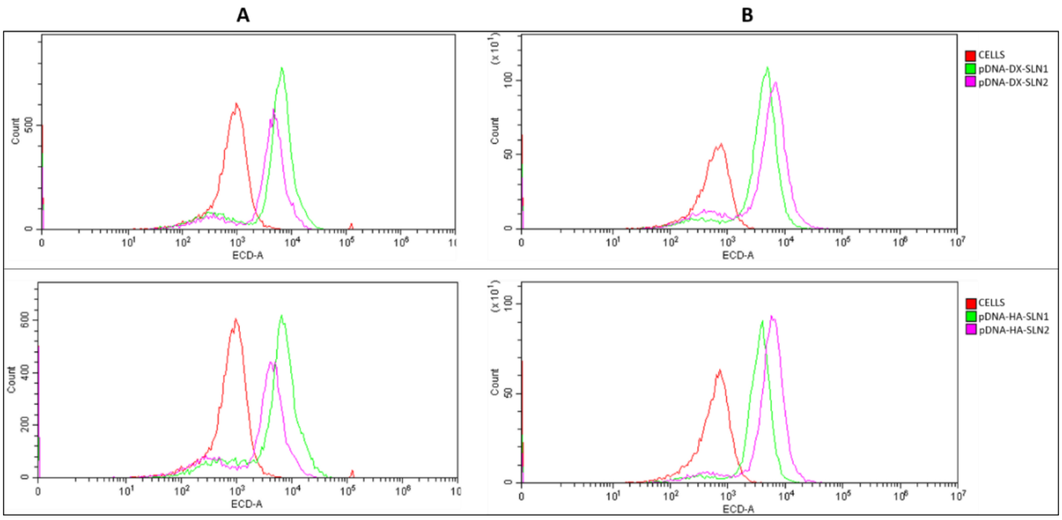

Figure S2. Flow cytometry analysis of cellular uptake of pDNA-vectors using Nile Red-labeled SLNs in ARPE-19. A: Intracellular uptake at 37 °C. B: Intracellular uptake at 4 °C. The higher the displacement to the right, the higher the fluorescence intensity in the cells. At 37° C both pDNA-DX-SLN1 and pDNA-HA-SLN1 were more displaced than those with SLN2. However, at 4° C, both pDNA-DX-SLN2 and pDNA-HA-SLN2 were more displaced to the right than those with SLN1.

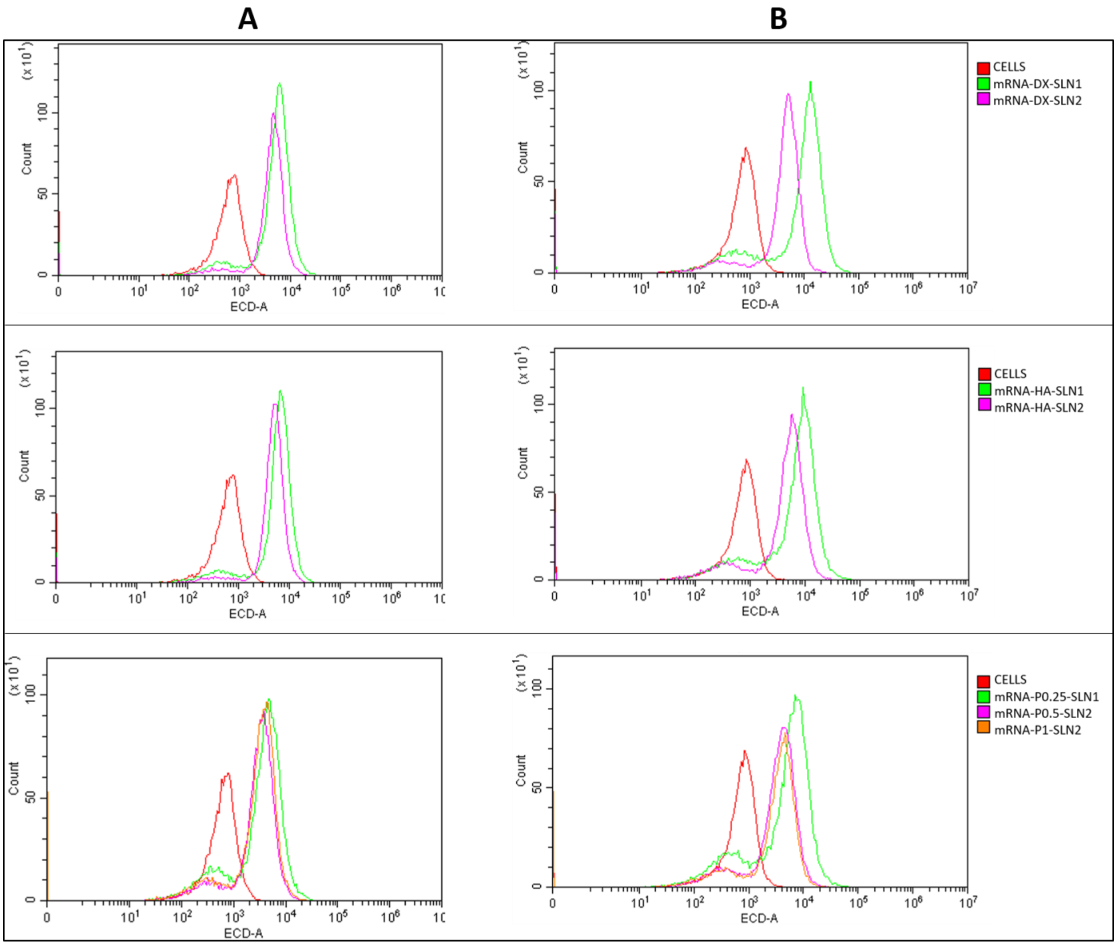

Figure S3. Flow cytometry analysis of cellular uptake of mRNA-vectors using Nile Red-labeled SLNs in ARPE-19. A: Intracellular uptake at 37 °C. B: Intracellular uptake at 4 °C The higher the displacement to the right, the higher the fluorescence intensity in the cells. At both 37 °C and 4 °C, all SLN1-based vectors the histograms were more displaced to the right than those formulated with SLN2.

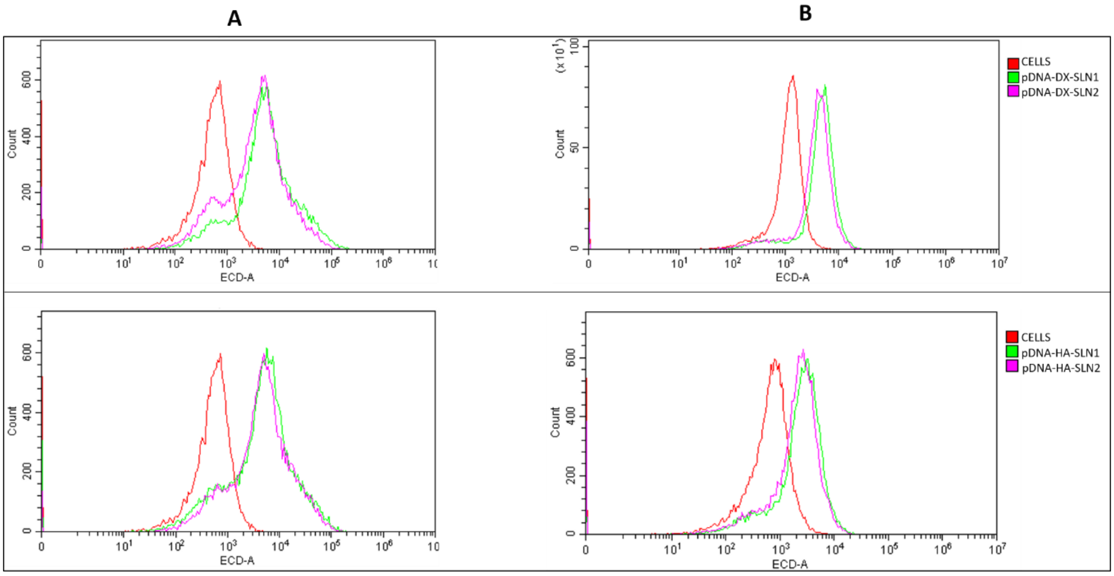

Figure S4. Flow cytometry analysis of cellular uptake of pDNA-vectors using Nile Red-labeled SLNs in HEK-293 cells. The higher the displacement to the right, the higher the fluorescence intensity in the cells. A: Intracellular uptake at 37 °C. B: Intracellular uptake at 4 °C. The displacement to the right was much higher at 37 °C than at 4 °C with all the vectors.

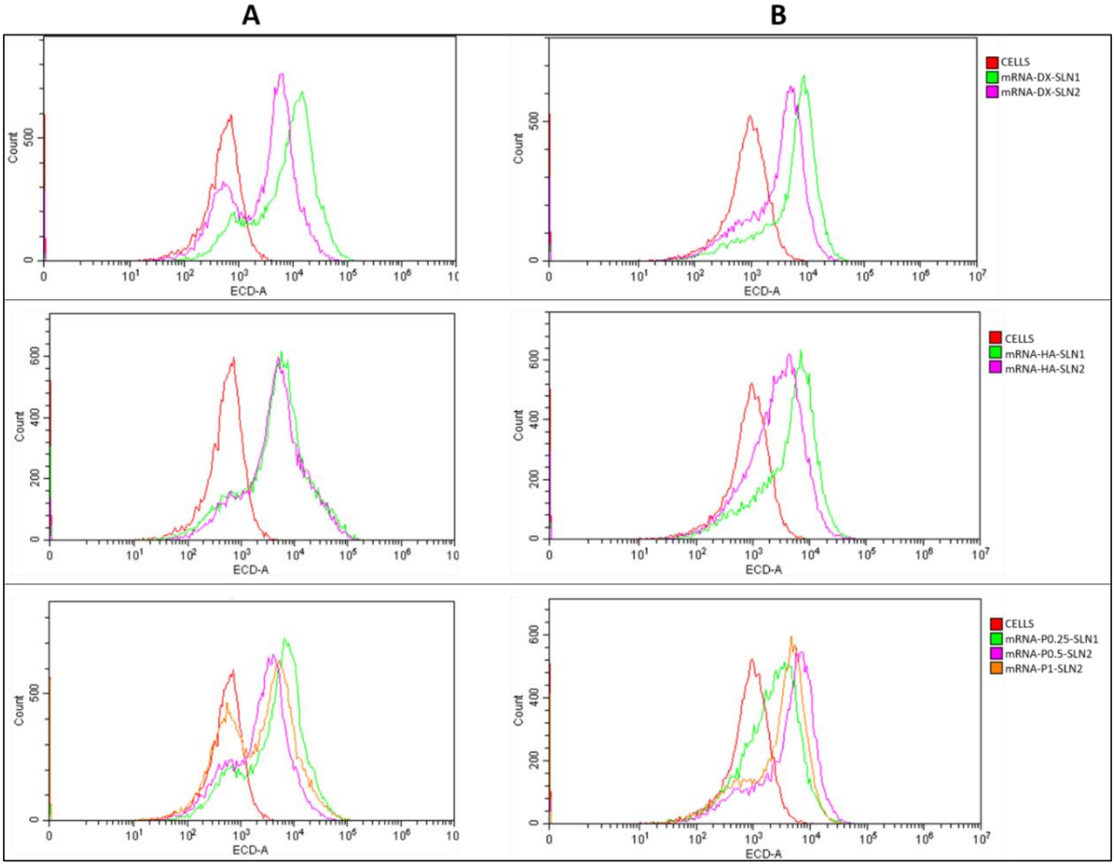

Figure S5. Flow cytometry analysis of cellular uptake of mRNA-vectors using Nile Red-labeled SLNs in HEK-293 cells. A: Intracellular uptake at 37 °C. B: Intracellular uptake at 4 °C. The higher the displacement to the right, the higher the fluorescence intensity in the cells. SLN1-based vectors were more displaced to the right than SLN2-based vectors at both temperatures, except mRNA-P0.5-SLN2 and mRNA-P1-SLN2 formulations at 4° C, which were more displaced than mRNA-P-SLN1.

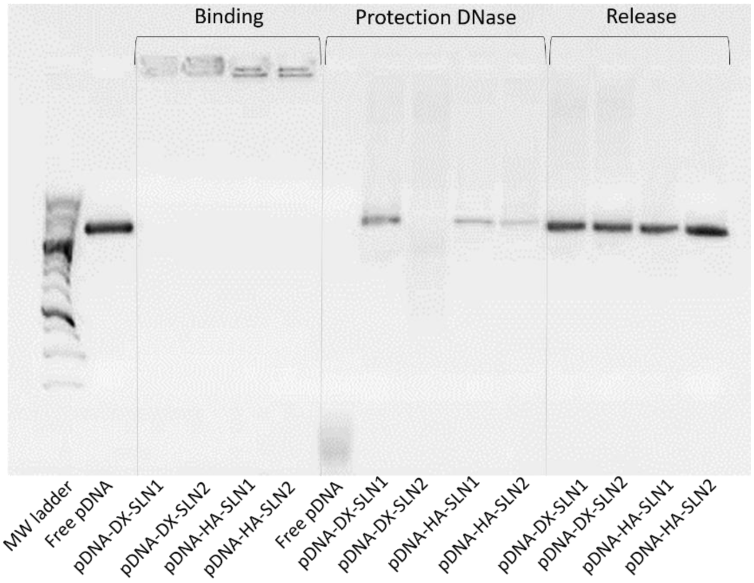

Figure S6. Study of the binding, protection and release of pDNA-based vectors formulated with SLN1 and SLN2 after 2 months of storage. It can be seen that at 2 months vectors were able to bind the pDNA, but the protection ability decreased respect to time 0.

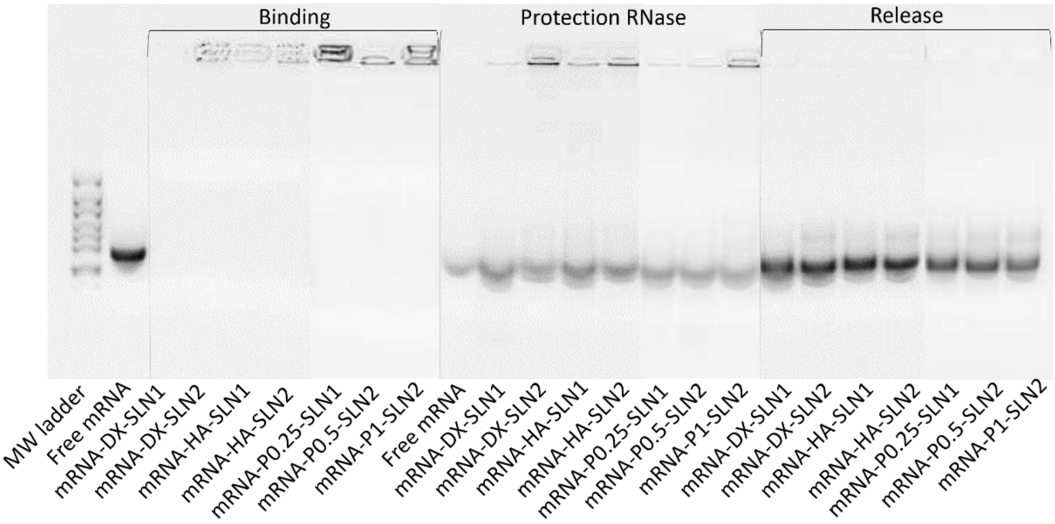

Figure S7. Study of the binding, protection and release of mRNA-based vectors formulated with SLN1 and SLN2 after 2 months of storage. It can be seen that at 2 months vectors were able to bind the mRNA, but the protection capacity decreased.

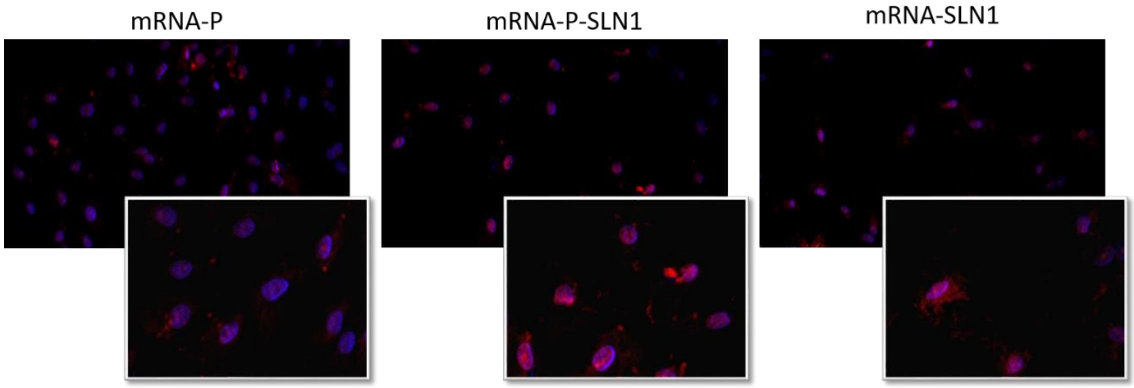

Figure S8. Microscopy images of the intracellular disposition in ARPE-19 cells of mRNA combined with P, SLN1 and the combination of both components. Images were captured with a Leica DM IL LED Fluo inverted microscope (Leica Microsystems CMS GmbH, Wetzlar, Germany). Red color: mRNA; blue color: cell nuclei. It can be seen that SLN1 alone did not condense the mRNA as much as P did.

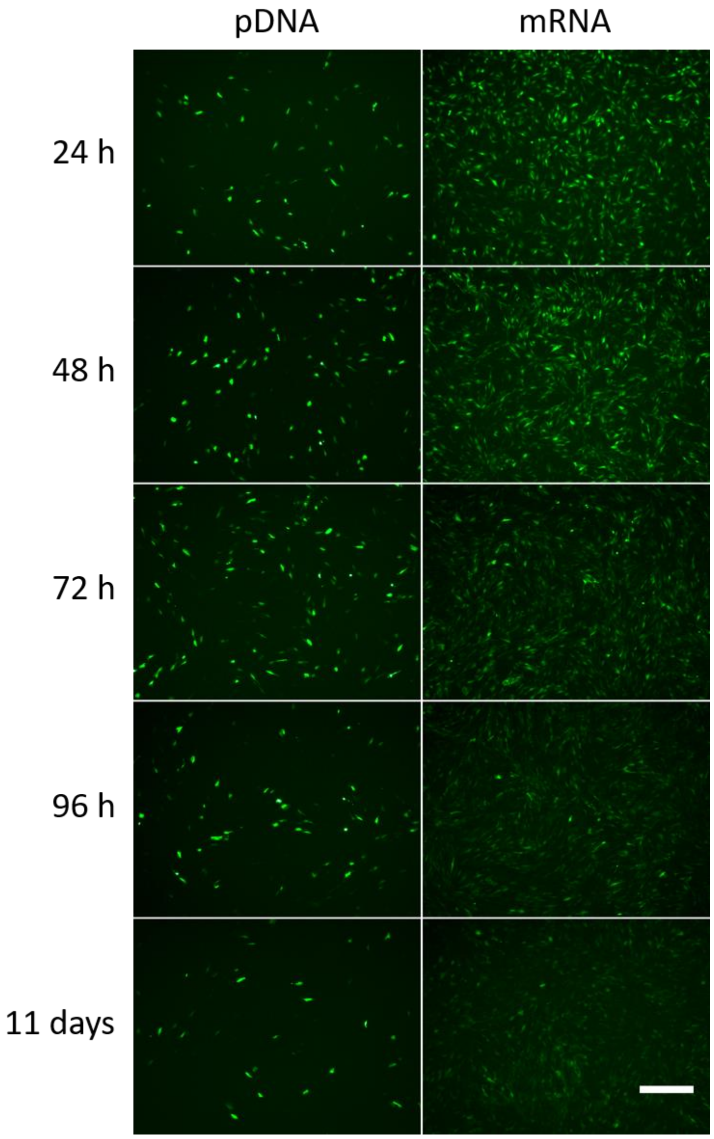

Figure S9. Microscopy images of ARPE-19 cells 24 h, 48 h, 72 h, 96 h and 11 days after transfection with pDNA-DX-SLN1 and mRNA-P0.25-SLN1 vectors. Green color corresponds to the GFP. Images were captured with a Leica DM IL LED Fluo inverted microscope (Leica Microsystems CMS GmbH, Wetzlar, Germany). Scale bar: 20 µm. mRNA formulations showed the highest transfection efficacy at 24-48 h, and the protein expression decreased notably at 96 h, whereas pDNA formulations showed the maximum transfection at 72-96 h. In both cases it lasted at least 11 days.

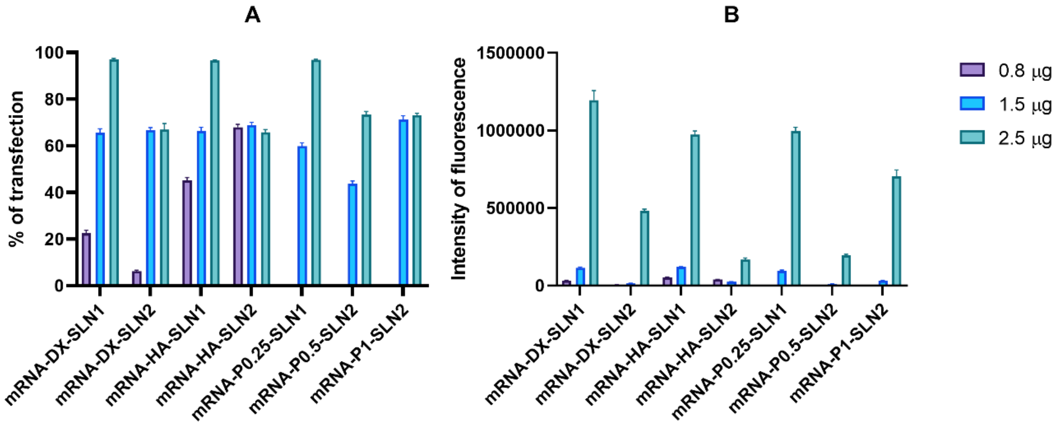

Figure S10. Optimization of the dose of mRNA-based vectors in ARPE-19 cells. A: percentage of transfected cells. B: Intensity of fluorescence of transfected cells. At the dose of 2.5 µg, vectors prepared with SLN1 showed transfection percentages of almost 100%. Therefore, this dose was discarded because it was difficult to optimize the formulations from such a high percentage. The dose of 0.8 µg in all cases gave very low percentages and intensities of transfection. Accordingly, the dose of 1.5 µg of mRNA was selected for the different studies carried out with freshly prepared vectors in ARPE-19 cells.
